# Modulation of Amyloidogenic Peptide Aggregation by Photoactivatable CO-Releasing Ruthenium(II) Complexes

**DOI:** 10.3390/ph13080171

**Published:** 2020-07-29

**Authors:** Daniele Florio, Maria Cuomo, Ilaria Iacobucci, Giarita Ferraro, Ahmed M. Mansour, Maria Monti, Antonello Merlino, Daniela Marasco

**Affiliations:** 1 Department of Pharmacy, University of Naples Federico II, 80134 Napoli, Italy; floriodaniele1@gmail.com (D.F.); cuomo.maria97@gmail.com (M.C.); 2 Department of Chemical Sciences, University of Naples Federico II, 80126 Napoli, Italy; ilaria.iacobucci@unina.it (I.I.); montimar@unina.it (M.M.); antonello.merlino@unina.it (A.M.); 3 CEINGE Biotecnologie Avanzate S.c.a r.l., University of Naples Federico II, 80145 Napoli, Italy; 4 Department of Chemistry Ugo Schiff, University of Florence, 50019 Sesto Fiorentino (FI), Italy; giarita.ferraro@gmail.com; 5 Department of Chemistry, Faculty of Science, University of Cairo, Gamma street, Giza 12613, Egypt; mansour@sci.cu.edu.eg

**Keywords:** modulators of amyloid peptide aggregation, metallodrugs, ruthenium(II) compounds, CO releasing molecules

## Abstract

Three Ru(II)-based CO-releasing molecules featuring bidentate benzimidazole and terpyridine derivatives as ligands were investigated for their ability to modulate the aggregation process of the second helix of the C-terminal domain of nucleophosmin 1, namely nucleophosmin 1 (NPM1)_264–277_, a model amyloidogenic system, before and after irradiation at 365 nm. Thioflavin T (ThT) binding assays and UV/Vis absorption spectra indicate that binding of the compounds to the peptide inhibits its aggregation and that the inhibitory effect increases upon irradiation (half maximal effective concentration (EC_50_) values in the high micromolar range). Electrospray ionization mass spectrometry data of the peptide in the presence of one of these compounds confirm that the modulation of amyloid aggregation relies on the formation of adducts obtained when the Ru compounds react with the peptide upon releasing of labile ligands, like chloride and carbon monoxide. This mechanism of action explains the subtle different behavior of the three compounds observed in ThT experiments. Overall, data support the hypothesis that metal-based CO releasing molecules can be used to develop metal-based drugs with potential application as anti-amyloidogenic agents.

## 1. Introduction

Ruthenium complexes represent an important class of metal-based agents with multiple potential applications in different diseases both in therapy and in diagnosis [1]. The antineoplastic function of ruthenium complexes is based on their interactions with DNA [2], mitochondria, and endoplasmic reticulum of cells [3,4], as well as with proteins involved in crucial cellular pathways [5]; they are able to induce tumor cell apoptosis, autophagy, and inhibition of angiogenesis [6,7]. Furthermore, they can be used as specific tumor biomolecular probes and as phototherapeutic agents [8].

In amyloid diseases [9,10], Ru(II)-complexes have been used for both diagnostic and therapeutic purposes [11]. In general, Ru(II) and Ru(III) complexes interact with peptides of different lengths and proteins forming adducts with different stoichiometries [5]. The formation of adducts with amyloidogenic peptides affects their toxicity and propensity to aggregate [11,12].

In pioneering studies, several well-known anticancer Ru(III) complexes, as NAMI-A [13] (imidazolium [trans-RuCl_4_(^1^H-imidazole)(dimethylsulfoxide-S)]), KP1019 [14] (indazolium [*trans*-RuCl_4_(^1^H-indazole)_2_]) and PMRU20 (2-aminothiazolium[trans-RuCl_4_(2-aminothiazole)_2_]), were investigated as in vitro inhibitors of Aβ_1−42_. It has been demonstrated that PMRU20 is highly effective in protecting cells from Aβ_1−42_ toxicity. Electrospray ionization-mass spectrometry (ESI-MS) analysis, evidenced the formation of adducts formed upon reaction of the compound with the peptide and thioflavin T assay, revealed that the presence of the Ru complex greatly reduces peptide aggregation in vitro [15]. Derivatives of NAMI-A and PMRU20 exhibit enhanced affinity toward Aβ_1–40_ [16]. The complex *fac*-[Ru(CO)_3_Cl_2_ (N^1^-thz)] (thz = 1,3-thiazole) binds the 1–28 fragment of the β−amyloid peptide [17]. A tetradentate Ru(II) complex containing a tris(2-pyridylmethyl)amine (tpa) ligand acts as inhibitor of Aβ_1-40_ aggregation in vitro [18]. Similar effects were observed in the context of human Islet Amyloid Polypeptide (hIAPP) amyloid process: NAMI-A, [Ru(bipy)Cl_4_] and [Ru(bipy)_2_Cl_2_] (bipy = 2,2ʹ-bipyridine) bind hIAPP, significantly altering its aggregative features [19]. The complex [Ru(bipy)(met)_2_]·3H_2_O (met = methionine) inhibits the fibril formation of hIAPP and depolymerizes mature fibrils [20]. Binuclear Ru complexes are able to interfere with hIAPP aggregation causing the formation of nanometric particles with reduced β-sheet content and cytotoxicity, exhibiting a greater affinity with respect to the corresponding mononuclear Ru complexes, likely due to synergic contribution of the second center acting as promising metallodrugs in the field of T2DM (Type 2 Diabetes Mellitus) [21]. A similar synergistic effect was shown by heterobimetallic Ru(II)−Au(I) complexes bearing different spacer lengths (4–8 polyethylene glycol units) able to bind to a fragment of the amyloid β_1−16_ in accordance with the binding abilities of parent drugs, RAPTA-C [22] and auranofin [23,24].

Recently, the use of luminescent transition metal complexes as non-conventional probes of amyloid formation has been also explored: the idea is to exploit their long-lived fluorescent life-times due to the spin–orbit coupling promoted by heavy metal ions [25]. For example, it has been shown that the luminescent dipyridophenazine (dppz) Ru(II) complex [Ru(bipy)_2_(dppz)]^2+^ can be used to monitor Aβ fibrillization. This compound is not photoluminescent in aqueous solution nor in the presence of monomeric Aβ, but it presents a strong photoluminescence in presence of Aβ aggregates. Indeed, the interaction of [Ru(bipy)_2_(dppz)]^2+^ with Aβ fibrils changes the polarity of the environment of the complex causing the enhancement of luminescence [26]. A similar behavior was exhibited by [Ru(dmbpy)(dcbpy)(dppz)] (dmbpy = 4,4′-dimethyl-2,2′-bipyridine, and dcbpy = 4,4′-dicorboxy-2,2′-bipyridine) [27]. More recently, it has been demonstrated that a Ru-based fluorescent probe, [Ru(phen)_2_(fipc)]^2+^ (phen = 1,10-phenanthroline, fipc = 5-fluoro-*N*-(1,10-phenanthrolin-5-yl)-1*H*-indole-2-carboxamide], is able to interact with Aβ in all forms (monomers, oligomers and fibrils) [28].

Overall, these data indicate that Ru compounds can be used alone or in combination with other metals to inhibit β-amyloid fibril formation in neurodegenerative diseases [29]. Among the Ru compounds to be used in this field, Ru(II) complexes that act as carbon monoxide-releasing molecules (CORMs) are particularly promising [30]. These molecules transport and release CO in controlled manner even if they are bound to proteins [31,32,33,34] and received particular attention as therapeutic agents for the anti-inflammatory [35] and anti-apoptotic [36] properties [37,38]. These compounds are promising for the treatment of neurodegenerative diseases since it has been shown that, at small concentration, CO can interfere with several crucial pathways in which dysregulation is at the basis of the disease development [39,40,41,42,43]. It has been also demonstrated that CORMs exhibit neuroprotective effects through a direct modulation of Aβ_1−42_ aggregation in vitro [44].

**Figure 1:**
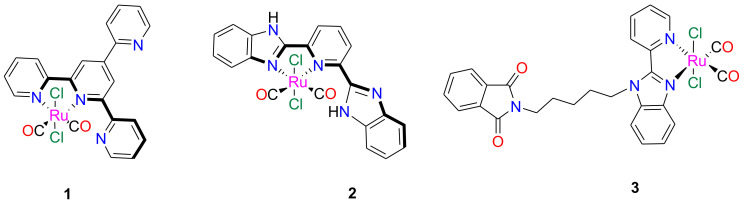
Chemical structures of the Ru(II)-based PhotoCORMs investigated in this study. CORMS = carbon monoxide-releasing molecules.

On the basis of these considerations, we think that Ru-based CORMs that can be photoactivated could represent a valuable strategy for the inhibition of amyloid peptide aggregation. Herein, we studied the ability of three photoinduced CO releasing, RuCl_2_(CO)_2_-based, compounds as inhibitors of aggregation of the peptide fragment corresponding to the helix H2 (residues 264–277) of C-terminal domain of nucleophosmin 1 (NPM1_264–277_). This peptide forms amyloid aggregates with β-sheet conformation and fibrillar morphology, toxic to neuroblastoma cells [45,46,47,48,49,50,51]. In particular, we chose three dicarbonyl Ru(II) compounds (Figure 1) functionalized with bidentate 4′-(2-pyridyl)-2,2′:6′,2″-terpyridine (1) [34], 2,6-bis(benzimidazole-2′-yl)pyridine (2) [52], and 1-(5-bromopentyl)phthalimide-2-(2’-pyridyl) benzimidazole (3) [53] ligands for this study.

Compound 3 exhibits interesting antifungal activity against *Candida albicans* and *Cryptococcus neoformans* in the micromolar range and is safe to the non-malignant HEK293 cell line [53]. Compounds **1**–**3** interact with proteins leading to the formation of adducts able to release CO upon UV irradiation at 365 nm [34,52,53].

## 2. Results and Discussion

### 2.1. NPM1_264–277_ Aggregation Is Suppressed by the Presence of the Investigated Ru(II) Complexes

**Figure 2:**
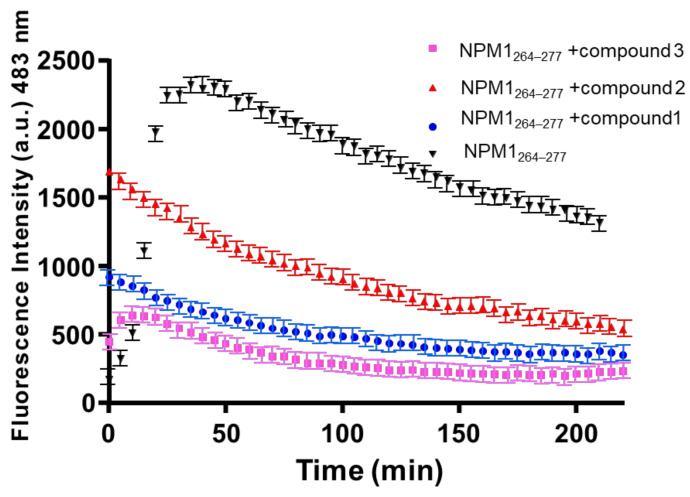
Overlay of time-courses of Thioflavin T (ThT) fluorescence emission intensity of nucleophosmin 1 (NPM1)_264–277_ in presence of the three Ru(II) compounds (**1**–**3**), at 1:1 peptide to metal-based compound molar ratio. Results are representative of two independent experiments.

The effects exerted by Ru(II) complexes on the self-aggregation process of NPM1_264–277_ were investigated through the analysis over time of Thioflavin T (ThT) fluorescence emission. In Figure 2, the overlay of fluorescence profiles for peptide alone and in the presence of the Ru compounds in 1:1 peptide:complex molar ratio is reported. As shown, the presence of all three metal complexes clearly induces a decrease of ThT signal when compared to that observed for the peptide alone. This finding suggests a modulation of peptide self-aggregation. At *t* = 0, the non–zero values and the different fluorescence intensities of ThT, in the presence of the three compounds, suggest a potential quenching/direct binding between metal complexes and the fluorescent probe [54]. At the early stage of aggregative process, compounds **1** and **2** already act as modulators of the aggregation, while compound **3** exhibits suppression of aggregation only after 20 min of stirring. Indeed, for compound **3** from *t* = 0 to *t* = 20 min, a slight increase of ThT signal is observable even though the enhancement is slower than that shown by the peptide alone and the ThT signal reaches intensities that are significantly lower than those found in the absence of complexes. A behavior similar to that reported for compound **3** has been observed for a Pt-compound that also acts as modulators of NPM1_264–277_ aggregation [55]. The different behavior of the three Ru compounds here analyzed as modulators of aggregation should be related to their structural features. Indeed, the higher hindrance of the phthalimide ligand in compound **3** could affect the kinetics of its potential interaction with the peptide; conversely, the presence of free pyridine or benzimidazole arm, ready to interact with the metal center, could facilitate the binding of compounds **1** and **2** to the peptide when compared to compound **3**. These features delay the compound **3** ability to modulate the aggregation of NPM1_264–277_ when compared to compounds **1** and **2**.

At greater interval time (~200 min), the ThT fluorescence value observed for NPM1_264–277_ in the presence of the three compounds is not far from the starting value found when the peptide is alone. This finding suggests that the compounds could interact with the monomeric form of NPM1_264–277_ hampering the self-recognition of different peptide chains.

**Figure 3:**
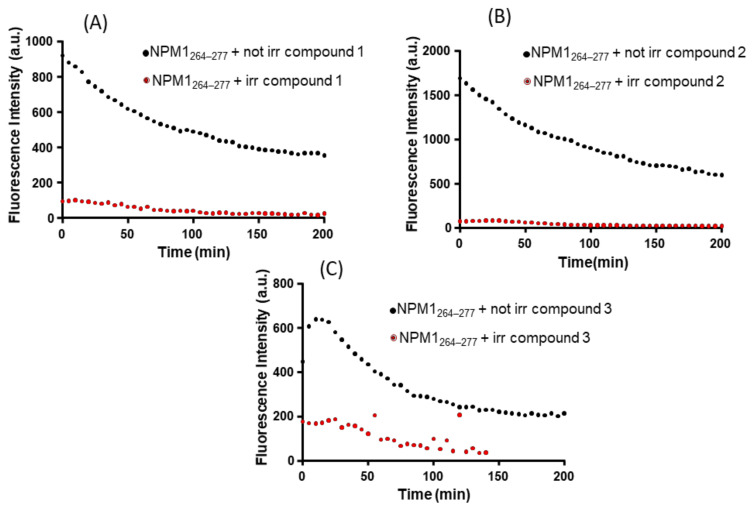
Overlay of time-courses of ThT fluorescence emission intensity of NPM1_264–277_ in presence of the three Ru(II) complexes: (**A**) compound **1**; (**B**) compound **2** (C) compound **3**, at 1:1 peptide to metal-based compound molar ratio. Results are representative of two independent experiments.

To evaluate if the compounds are able to modulate the aggregation process of NPM1_264–277_ upon releasing of CO, the same ThT time-courses were registered using Ru(II) compounds that have been irradiated with a UV lamp at 365 nm. A comparison of the behavior of irradiated and non-irradiated compounds is reported in Figure 3. The analysis of reported ThT profiles indicates that the investigated PhotoCORMs reduce the amyloid aggregation more strongly after illumination. The value of ThT fluorescence intensity observed for the samples of the peptide in the presence of the irradiated complexes is significantly lower than that observed for non-irradiated complexes. After irradiation, compound **1** also provides a faster inhibitory effect than the corresponding non-irradiated sample, as inferable from normalized signals reported in Figure S3. This may be explained considering that compound **1** releases CO faster than compounds **2** and **3**, as previously discussed [34,52,53], under the same experimental conditions.

### 2.2. The Ligand Field of Ru(II) Complexes Changes in the Presence of NPM1_264–277_

Since it is known that irradiation with UV light induces release of CO from the investigated Ru-based PhotoCORMs [34,52,53] and considering that the irradiated complexes act as modulators of the aggregation process of the amyloid peptide more efficiently than non-irradiated samples, we supposed that the mechanism at the basis of the activity of the compounds was grounded on the exchange of CO with a side chain of a peptide residue. To prove this hypothesis, UV-Vis absorption spectroscopy and electrospray mass spectrometry data have been collected.

The ability of NPM1_264–277_ to perturb ligand fields of investigated CORMs was analyzed through UV/Vis absorption spectroscopy. The lack of changes in the position of Ligand to Metal Charge Transfer (LMCT) or Metal to Ligand Charge Transfer (MLCT) bands indicates a retention of symmetry around metal center, while the variations of the position and intensity of the maximum absorption wavelength suggest possible substitutions of the ligands in the coordination of Ru(II) by side chains of residues of the peptide. Since the sequence of the peptide is VEAKFINYVKNCFR, the side chains that can coordinate the Ru center are C, E, N, K, or R.

**Figure 4:**
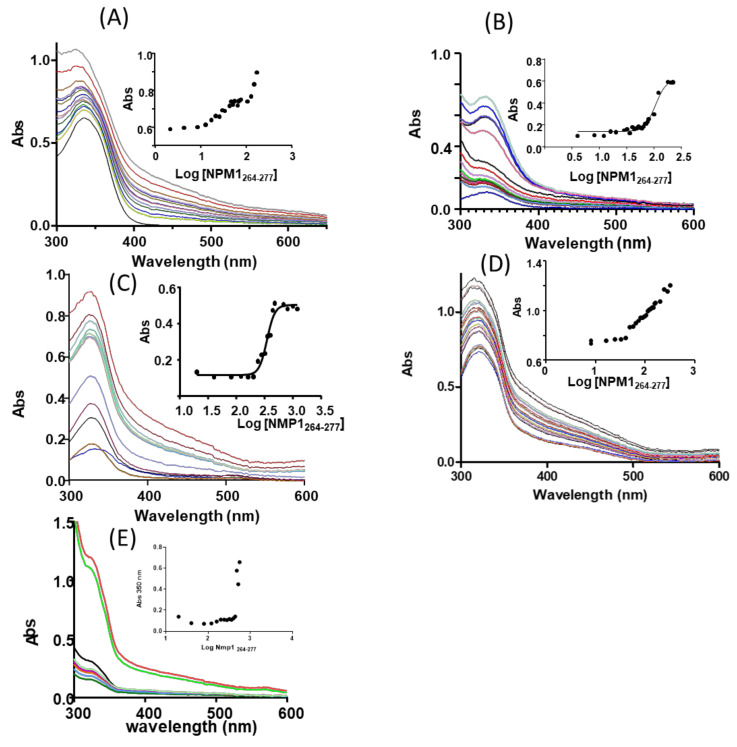
Absorption spectra of (**A**,**B**) compound **2**, (**C**,**D**) compound **3** and (**E**) compound **1** in UV irradiated (**B**,**D**) and not irradiated (**A**,**C**,**E**) preparation, upon the addition of increasing amount of NPM1_264–277_ . As insets UV intensities at ~330 nm versus Log concentration of NPM1_264–277_ have been reported.

The spectra of the three Ru(II) complexes registered upon the addition of increasing amount of NPM1_264–277_ are reported in Figure 4. In aqueous solutions, all three compounds present MLCT bands (at ~330 nm) with a hypsochromic shift with respect to the value observed in pure Dimethyl Sulfoxide (DMSO) (data not shown and references [34,52,53]), in agreement with what expected on the basis of previous analyses [34]. In this respect, it should be underlined that the metal compounds are soluble and stable for 24 h in the analyzed aqueous solutions (data not shown [34,52,53]).

The addition of NPM1_264–277_ to the Ru compounds produces in all the cases an increase of intensity of the signal. Unfortunately, due to poor solubility of the adduct formed upon reaction of compound **1** with the peptide, it was not possible to reach saturation state when the sample is not irradiated (Figure 4E), and it was not possible to collect spectra when the sample is irradiated.

Compound **2** does not reach saturation (Figure 4A), while for compound **3** (Figure 4C), the addition of peptide allows to reach saturation at 1:3 complex: peptide molar ratio. For compound **3**, the titration allows a fit of experimental data with a saturated profile, providing an estimation of EC_50_ (half maximal effective concentration) = 345.4 ± 1.0 µM (inset of Figure 4C).

In the case of irradiated samples, only compound **2** reaches saturation at complex: peptide molar ratio of 1:4. For compound **2**, an EC_50_ = 105.3 ± 1.1 µM was estimated (inset of Figure 4B).

### 2.3. ESI-MS Analysis of NPM1_264–277_ in the Presence of the Ru Compounds

Despite samples of NPM1_264–277_ in the presence of the three Ru compounds were prepared and analyzed through ESI-MS, spectra have been successfully registered only in the case of the adducts formed upon reaction of compound 2 with the peptide.

**Figure 5:**
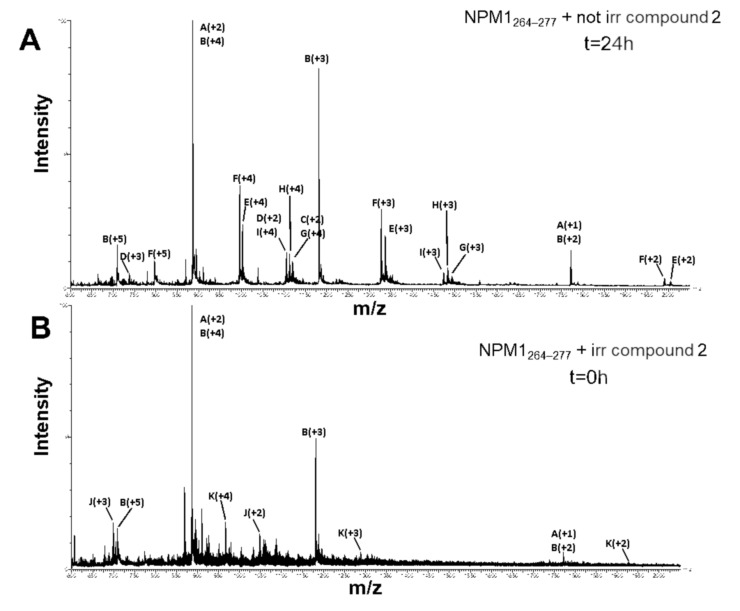
ESI-MS multi-charged spectra of NPM1_264–277_ +compound 2 after 24 h of incubation (no irradiation) (**A**) and UV-irradiation upon freshly prepared sample (**B**).

ESI-MS analysis of NPM1_264–277_ following incubation with compound 2 shows the presence of NPM1_264–277_ both as monomer (1770.93 ± 0.01 Da) and as disulfide-bridged dimer (3541.19 ± 0.95 Da). Both species generate adducts with compound 2 either spontaneously or after UV irradiation; for each metal compound that is bound to the peptide, a trifluoroacetate (TFA) ion, coming from the background solvent, is present. As reported in Figure 5A and Table 1, both monomeric and dimeric forms of NPM1_264–277_ binds one molecule of compound 2 losing two chloride ions, as demonstrated by the presence of two species of 2236.37 ± 0.94 Da and 4005.54 ± 0.93 Da molecular weight, respectively. This finding suggests a bidentate mode of binding of the Ru-containing fragment to the peptide. Moreover, the NPM1_264–277_ dimer also forms an adduct with two molecules of the metal compound (4470.40 ± 0.53 Da). In addition, metal compound species lacking one carbon monoxide molecule can also bind the peptide (Table 1). As demonstrated by ESI-MS analysis of the non-irradiated sample, the release of CO in the presence of the peptide is a process independent on irradiation. However, as expected, it is increased by UV treatment. Indeed, as reported in Figure 5B and Table 2, ESI-MS spectra display the occurrence of adducts formed by exchange of both chloride ions and one CO molecule, after shorter incubation time. It is important to highlight that following irradiation, the CO loss from the complex occurs in a complete way. The finding that the NPM1_264–277_ dimer binds the same Ru-containing fragments when compared to the monomer suggests that the metal center does not coordinate the cysteine residue. Thus, E, N, K, or R are the only residues of the peptide that can be involved in the coordination of the metal containing fragment.

## 3. Materials and Methods

### 3.1. Peptide and Metal Compound Synthesis

Acetylated and amidated peptide corresponding to NMP1_264–277_ sequence (VEAKFINYVKNCFR) was synthesized as already reported [56]. Reagents for Solid-Phase Peptide Synthesis (SPPS)were purchased from Iris Biotech (Marktredwitz, Germany) and solvents from Romil (Dublin, Ireland). The peptide was treated with Hexafluoro-2-propanol (HFIP) (at 50% (*v*/*v*) in water), purified by RP-HPLC and identified through LC-MS. The Ru(II) complexes (Figure 1) were synthetized as previously described [34,52,53]. UV- irradiation was performed using a UV/vis hand lamp (365 nm, filter 145 × 48 nm, power 2 × 6 Watt, Vilber lourmat, France). In experiments with irradiated complexes, the metal compounds were irradiated, and, only successively, the peptide and, finally, ThT (for fluorescence assay) were added.

### 3.2. ThT Fluorescence

The abilities of Ru(II) complexes to interfere with amyloid aggregation of NMP1_264–277_ through the analysis of time-courses of thioflavin (ThT) fluorescence, at 25 °C using a Jasco FP 8300 spectrofluorometer (Tokyo, Japan), (settings: λ_exc_ = 440 nm, λ_em_ = 483 nm). Peptide and ThT concentrations were 100 and 50 µM, respectively, 10 mM borate buffer at pH = 9.0, DMSO ~0.2%. Control experiments indicate that the fluorescence spectra of the metal compounds in the presence of ThT show negligible intensities. The analysis was carried out maintaining a magnetic stirring in a 10 mm path-length quartz cuvette. Data were reported as mean of two independent experiments.

### 3.3. UV/Vis Spectroscopy

UV/Vis spectra of the Ru(II) compounds titrated with NMP1_264–277_ were registered with a Nanodrop 2000 c of Thermo Scientific (Waltham, MA, USA) at the same temperature and buffer conditions of fluorescence assay. Titrations were carried out at a fixed concentration of the Ru(II) complexes (~400 µM for not irradiated and ~80 µM for irradiated compounds), gradually increasing the equivalents of the peptide through sequential addition of 1.0 µL of different stock solutions (2, 10 mM) in water, kept at 0 °C. Each spectrum was registered (300–500 nm) upon the addition of the peptide, after a stirring time of 2 min. EC_50_ value was derived from non-linear regression of the data employing log (inhibitor) vs. response and “dose-response stimulation equation” of GraphPad program [57].

The solution stability of the three compounds in different buffers has been evaluated by collecting the UV/Vis spectra of the compounds in the presence of 10 mM sodium citrate (pH = 5.1), sodium acetate (pH = 6.8) and sodium phosphate (pH = 7.4) over 24 h. The compounds have been dissolved in DMSO and then added to the selected buffers to reach a final concentration of 50 μM. The DMSO final concentration is less than 0.5% (*v*/*v*). UV/Vis spectra were registered on a Varian Cary 5000 UV-Vis spectrophotometer at room temperature, using 1 cm path length cuvettes and the following parameters: 300–500 nm range, 200 nm/min, 2.0 nm bandwidth. The photoactivable CO-release properties of the compounds have been also analyzed by following the changes in the UV/Vis spectral profiles before and after 20 min of irradiation at 365 nm (see, for example, Figure S1 and Figure S2, where spectra of compound 1 are reported).

### 3.4. ESI-MS Analysis

Solutions of NPM1_264–277_, at a concentration of 100 µM in 10 mM borate buffer at pH = 9, incubated with compound 2 in a molar ratio of 1:10 for 0 h and 24 h with and without UV irradiation, respectively, were diluted in ammonium acetate 15 mM and analyzed by ESI-MS, as described in a previous work [56]. Raw mass spectra were processed by MaxEnt3 algorithm (MassLynx4.1, Micromass, Waters; Milford, MA, USA).

## 4. Conclusions

In this study we have carried out a biophysical investigation on the effect of three photoinduced Ru(II) dicarbonyl complexes on the self-aggregation of an amyloid model peptide, before and after irradiation at 365 nm. Spectroscopic studies reveal that the three Ru compounds exhibit a strong modulatory effect on the amyloid-like aggregation of NMP1_264–277_, both in the dark and upon the illumination.

ThT fluorescence time courses experiments indicate that the three compounds have a different behavior: compounds **1** and **2**, functionalized with bidentate ligands, incorporating free coordination site close to the metal ion, promptly reduce the aggregation process, while compound **3**, which does not incorporate close donor site, needs more time to induce reduction of aggregation. Electrospray ionization mass spectrometry and UV/Vis absorption spectroscopy experiments suggest that the mechanism of the modulation of aggregation is related to the formation of adducts formed upon reaction of the metal-based compounds with the peptide, through the binding of the peptide to the coordination sphere of Ru(II) ion that follows the release of the two coordinated Cl ions and one CO ligand. This mechanism of action explains why irradiated complexes are better than the non-irradiated compounds in modulating the aggregation of the amyloid sequence and provides a rationale to explain the different behavior of the three compounds observed in the ThT measurements: the absence of closer free coordination sites to Ru(II), and consequently the slower CO release, probably affects the kinetics of the formation of the Ru-fragment/peptide adduct causing a delay in its ability to modulate the aggregation of the peptide. On the other hand, the faster CO release of compound **1**, because of the presence of closely coordination-able free pyridyl arm, led to an improvement in the kinetics of inhibition in its irradiated form when compared to the non-irradiated molecule. This result suggests that a fine tuning of metal ligands could significantly affect the anti-amyloidogenic properties of metallodrugs and highlights the importance of a structural characterization of the adducts formed by reaction of metallodrugs with amyloid peptides.

Our data indicate that the investigated compounds are able to modulate the aggregation process of amyloid models in vitro and suggest an inhibitory mechanism that, however, needs future microscopy investigations to be confirmed.

In conclusion, the results here reported offer a valid support to the idea that photoactivatable metal carbonyl-based compounds may be successfully exploited or the treatment of neurodegenerative diseases.

## Figures and Tables

**Table 1:** ESI-MS detected species formed by NPM1_264–277_ and compound **2** at 24 h of incubation. The experimental *m*/*z* values and their charge, the experimental and theoretical molecular weight, and the corresponding species are reported. TFA: trifluoroacetate.

*m*/*z* Signal	Charge	Experimental MW	Theoretical MW	Species
886.47 1771.93	A + 2 A + 1	1770.93 ± 0.01	1770.91	Monomer (NPM1_264–277_(M))
708.83 885.97 1181.63 1771.93	B + 5 B + 4 B + 3 B + 2	3541.19 ± 0.95	3539.82	Dimer (NPM1_264–277_(D))
746.78 1118.72	C + 3 C + 2	2236.37 ± 0.94	2234.27	NPM1_264–277_(M) + 1 compound **2** – 2 HCl + 1 TFA
736.65 1104.48	D + 3 D + 2	2207.28 ± 0.48	2206.26	NPM1_264–277_(M) + 1 compound **2** – 2 HCl – 1 CO + 1 TFA
1002.23 1335.97 2004.43	E + 4 E + 3 E + 2	4005.54 ± 0.93	4003.16	NPM1_264–277_(D) + 1 compound **2** – 2 HCl + 1 TFA
796.18 994.48 1326.63 1988.96	F + 5 F + 4 F + 3 F + 2	3975.64 ± 1.08	3975.15	NPM1_264–277_(D) + 1 compound **2** – 2 HCl – 1 CO + 1 TFA
1118.47 1491.32	G + 4 G + 3	4470.40 ± 0.53	4466.55	NPM1_264–277_(D) + 2 compound **2** – 4 HCl + 2 TFA
1111.71 1481.64	H + 4 H + 3	4442.35 ± 0.46	4438.49	NPM1_264–277_(D) + 2 compound **2** – 4 HCl – 1 CO + 2 TFA
1104.23 1471.97	I + 4 I + 3	4412.90 ± 0.01	4410.48	NPM1_264–277_(D) + 2 compound **2** – 4 HCl – 2 CO + 2 TFA

**Table 2:** ESI-MS detected species formed by NPM1_264–277_ with irradiated compound **2** at 0 h of incubation. The experimental *m/z* values and their charge, the experimental and theoretical molecular weight, and the corresponding species are reported.

*m*/*z* Signal	Charge	Experimental MW	Theoretical MW	Species
886.48 1772.00	A + 2 A + 1	1770.97 ± 0.02	1770.91	Monomer (NPM1_264–277_(M))
709.39 885.99 1180.99 1771.49	B + 5 B + 4 B + 3 B + 2	3540.68 ± 0.81	3539.82	Dimer (NPM1_264–277_(D))
698.31 1046.99 2093.92	J + 3 J + 2 J + 1	2092.28 ± 0.68	2093.23	NPM1_264–277_(M) + 1 compound **2** – 2 HCl – 1 CO
644.94 773.39 966.48 1288.99 1932.73	K + 6 K + 5 K + 4 K + 3 K + 2	3862.96 ± 0.89	3862.13	NPM1_264–277_(D) + 1 compound **2** – 2 HCl – 1 CO

## Supplementary Materials

The following are available online at https://www.mdpi.com/1424-8247/13/8/171/s1.
