# Supplementary material for: Modulation of Amyloidogenic Peptide Aggregation by Photoactivatable CO-Releasing Ruthenium(II) Complexes
Source: Pharmaceuticals (Basel). 2020 Jul 29;13(8):171. doi: 10.3390/ph13080171 (PMC7464691; doi:10.3390/ph13080171)
Supplement: Supplementary file 1 [file pharmaceuticals-13-00171-s001.pdf]

pharmaceuticals

SUPPORTING INFO

Molecular Inhibition of Amyloidogenic Peptide Aggregation by Photoactivatable CO-Releasing Ruthenium(II) Complexes

Daniele Florio, Maria Cuomo, Ilaria Iacobucci, Giarita Ferraro, Ahmed M. Mansour, Maria Monti, Antonello Merlino and Daniela Marasco

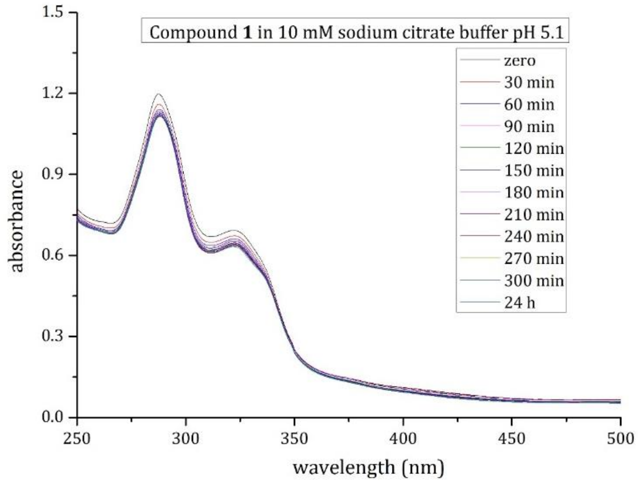

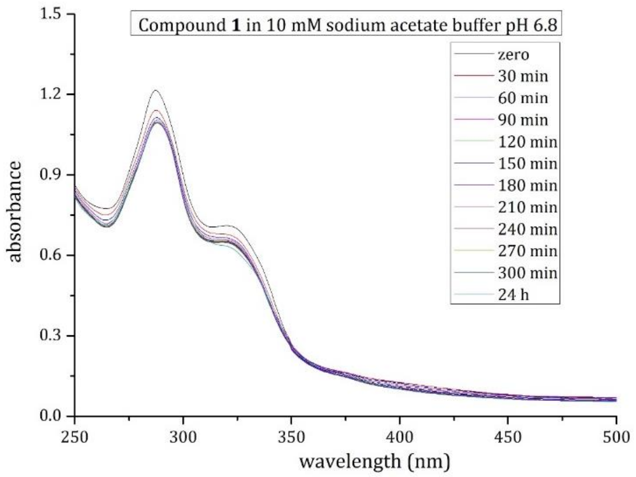

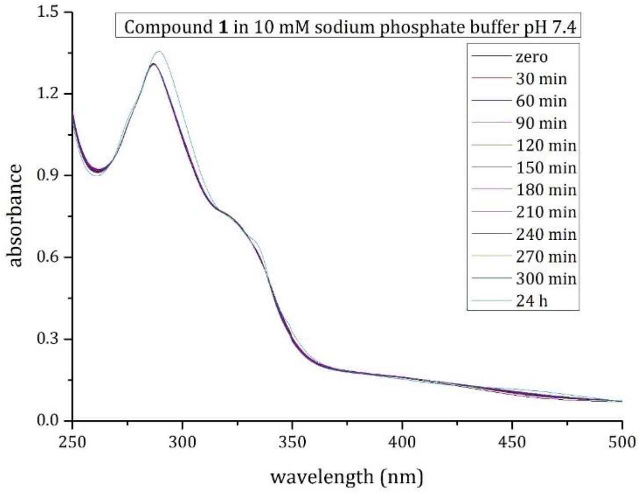

Figure S1. UV-vis time course of 420 in 10 mM sodium citrate at pH 5.1, in 10 mM ammonium acetate at pH 6.8 and in 10 mM sodium phosphate at pH 7.4 over 24 h. UV-vis spectroscopy has been also used to evaluate the CO-release upon irradiation as previously reported by Mansour and co-workers. The spectra have been registered before and after 20 min of irradiation with a 6W UV lamp. In agreement with previous suggestions, upon 20 min exposure to light at 365 nm, the spectra of the compound slightly change, suggesting the loss of CO and the possible repositioning of the terpyridine ligand from a bidentate to a tridentate mode of binding to the metal center. Irradiation of 420 in sodium citrate, ammonium acetate and sodium phosphate buffers produces a blue shift of the λmax from 289 nm to 285 nm, the disappearance of the 325 nm band and the appearance of a new low intensity band at 390 nm.

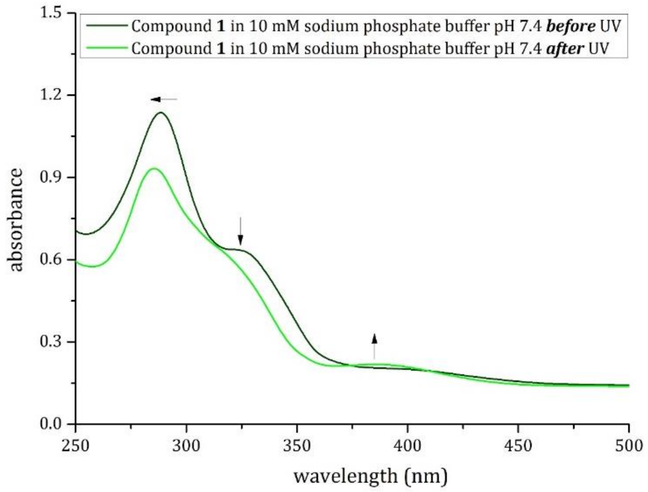

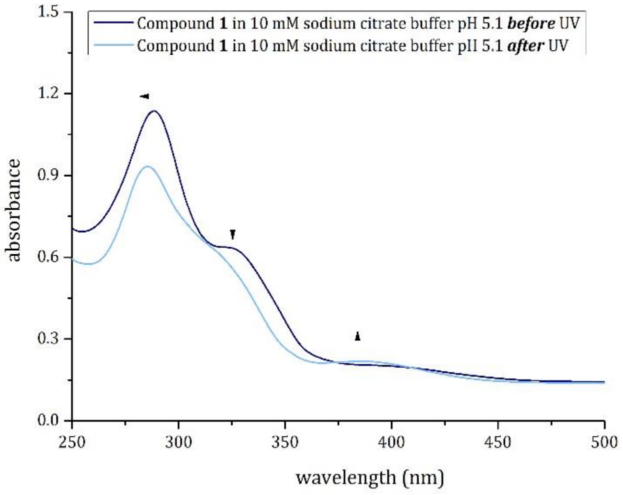

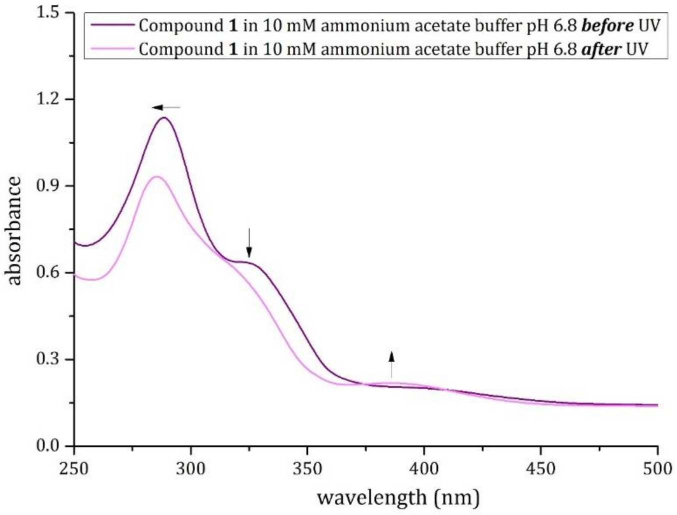

Figure S2. UV-vis spectra of 420 collected before and after 20 min of irradiation at 365 nm in 10 mM sodium citrate at pH 5.1, in 10 mM ammonium acetate at pH 6.8 and in 10 mM sodium phosphate at pH 7.4.

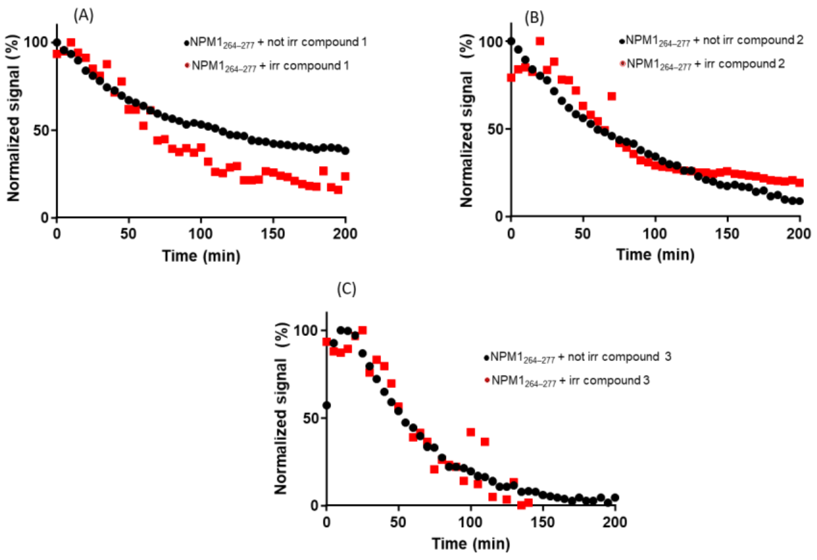

Figure S3. Overlays of time-courses of normalized ThT signals for NPM1264-277 in presence of Ru(II) compounds in UV-irradiated and not irradiated samples of (A) compound 1, (B) compound 2, (C) compound 3.
